# Deciphering the Mechanism of Action of the Antimicrobial Peptide BP100

**DOI:** 10.3390/ijms25063456

**Published:** 2024-03-19

**Authors:** Gerard Riesco-Llach, Sergi Llanet-Ferrer, Marta Planas, Lidia Feliu

**Affiliations:** LIPPSO, Department of Chemistry, Campus Montilivi, Universitat de Girona, 17003 Girona, Spain; gerard.riesco@udg.edu (G.R.-L.); sergillanet@gmail.com (S.L.-F.)

**Keywords:** membrane interaction, spectroscopic techniques, biophysical studies, MD simulations, carpet-like mechanism

## Abstract

The linear undecapeptide KKLFKKILKYL-NH_2_ (BP100) highlights for its antibacterial activity against Gram-negative bacteria and its low toxicity. These excellent biological properties prompted the investigation of its mechanism of action, which were undertaken using spectroscopic techniques, biophysical analysis, microscopy, and molecular dynamic simulations. Studies were conducted in different membrane environments, such as anionic, zwitterionic, and mixed membranes, as well as in vesicles (LUVs and GUVs) and bacteria. The findings suggest that BP100 exhibits a preference for anionic membranes, and its mechanism of action involves charge neutralization and membrane permeabilization. In these membranes, BP100 transitions from an unstructured state in water to an α-helix with the axis parallel to the surface. MD simulations suggest that after electrostatic interaction with the membrane, BP100 flips, facilitating the insertion of its hydrophobic face into the membrane bilayer. Thus, BP100 adopts an almost vertical transmembrane orientation with lysine side chains snorkelling on both sides of the membrane. As a result of the rotation, BP100 induces membrane thinning and slow lipid diffusion and promotes water penetration, particularly in anionic lipid membranes. These investigations pointed towards a carpet-like mechanism and are aligned with the biological activity profile described for BP100. This review covers all the studies carried out on the mechanism of action of BP100 published between 2009 and 2023.

## 1. Introduction

The widespread and often inappropriate use of antibiotics, both in human healthcare and agriculture, has accelerated the emergence of resistant bacteria worldwide [1]. This increasing occurrence of antibacterial resistance, along with the transmission of resistant bacterial strains among humans, animals, and their environment, has contributed to a continuous rise in the number of deaths from multidrug-resistant infections. About 4.95 million deaths in 2019 were related to antibacterial resistance, and 1.27 million people died directly from the effects of resistant bacteria [2]. Moreover, the number of effective antibiotics currently available is becoming more and more limited. All these considerations underline bacterial resistance as a huge threat to public health, with hospital- and community-acquired infections becoming significantly more challenging to treat. Therefore, the development of new antibiotics, especially those with innovative mechanisms, is currently a crucial priority in public health.

There is also an urgent need for new antimicrobials useful in agriculture because pests and plant diseases cause a huge impact on crop production. It is estimated that up to 40% of crops are lost annually, which supposes a cost of more than USD 290 billion per year. Today’s increasing globalisation is favouring pest movement and transmission, therefore significantly contributing to these losses. Moreover, in the coming years, climate change is expected to worsen this problem because the increasing temperatures may favour the spread of crop pests and diseases [3]. Nowadays, the main strategies for pest management consist of conventional agricultural methods, such as sanitary pruning, and agrochemicals, including antibiotics. However, these strategies have numerous limitations. In particular, conventional cultural practices are often ineffective. Regarding agrochemicals, despite their efficacy, they may have negative consequences on the environment and antibiotics can lead to the development of antimicrobial resistance. At the same time, consumers are becoming more and more aware of these problems and demand safer and more environmentally friendly agricultural practices. In this context, the authorities are imposing restrictions on the use of antibiotics, aligned with the United Nations Development Goals, the European Green Deal, and the Farm to Fork strategy. Therefore, nowadays, there is a lack of efficient and ecofriendly compounds to treat plant diseases.

The bacterial membrane constitutes one of the potential targets of antibiotics. Since it is essential for bacterial survival, being involved in numerous cellular functions, its permeabilization and disruption ultimately leads to cell death. Compounds able to tackle bacterial membranes are unlikely to promote antibacterial resistance, because multiple mutations in the membrane composition would be necessary, which would require a long time. Therefore, compounds with a direct activity on the bacterial membrane are considered a good alternative to conventional antibiotics. Antimicrobial peptides fulfil this condition, their primary mechanism being the damage of the bacterial membrane [4,5,6,7,8,9]. These peptides also possess additional features that render them as suitable candidates as antibacterial agents. Antimicrobial peptides are present in all forms of life and play an important role in the immune system of plants and animals to defend themselves against pathogens. They display a broad spectrum of action against a variety of bacteria, fungi, and viruses. Their structural characteristics are crucial for their mechanism of action, in particular, their cationicity, amphiphilicity, and hydrophobicity. Their positive charge ranges from +2 to +9 and contributes to their electrostatic attraction with the anionic bacterial membranes. Their amphipathic character and their hydrophobicity facilitate their insertion into the lipidic bilayers. In general, antimicrobial peptides are unstructured in an aqueous environment and upon membrane binding adopt a secondary structure, for example, an α-helix. Then, membrane disruption occurs through different models, including carpet, barrel-stave, or toroidal pore. In addition, it has been described that antimicrobial peptides may interact with intracellular targets after crossing the membrane.

For the last 20 years, our research has focused on the search for antimicrobial peptides with activity against economically important Gram-negative plant pathogens, including *Erwinia amylovora* as well as *Pseudomonas* and *Xanthomonas* species. In 2006, we identified the linear undecapeptide KKLFKKILKYL-NH_2_ (BP100) with high activity against these bacteria, while its haemolysis and phytotoxicity were low [10]. To further optimize the biological profile of this lead peptide, we designed and synthesized BP100 analogues through the incorporation of d-, triazolyl, biaryl, or acylated amino acids, or even through its conjugation with a plant defence elicitor peptide or with another antimicrobial peptide [11]. We also designed peptide conjugates derived from BP100 for their production in plant systems, i.e., in rice seeds [12,13]. Instigated by the excellent biological profile of BP100, in parallel, other research groups have reported new applications, such as anticancer activity, antibacterial activity against Gram-positive strains or cell-penetrating peptide properties [14,15,16,17,18,19]. BP100-coated gold or alumina nanoparticles have also been described as drug delivery systems or antibacterial agents for medical applications [20,21,22].

Based on the excellent biological properties of BP100, research involving different fields including biophysics, spectroscopy, and molecular dynamics (MD) simulations has been performed to shed light on the mechanism of action of this antimicrobial peptide. Herein, all these works were reviewed from the first one published in 2009 to the latest ones in 2023. This review also covers studies on BP100 derivatives that contribute to understanding the mechanism of action of BP100.

## 2. Mechanistic Studies on BP100

**Figure 1:**
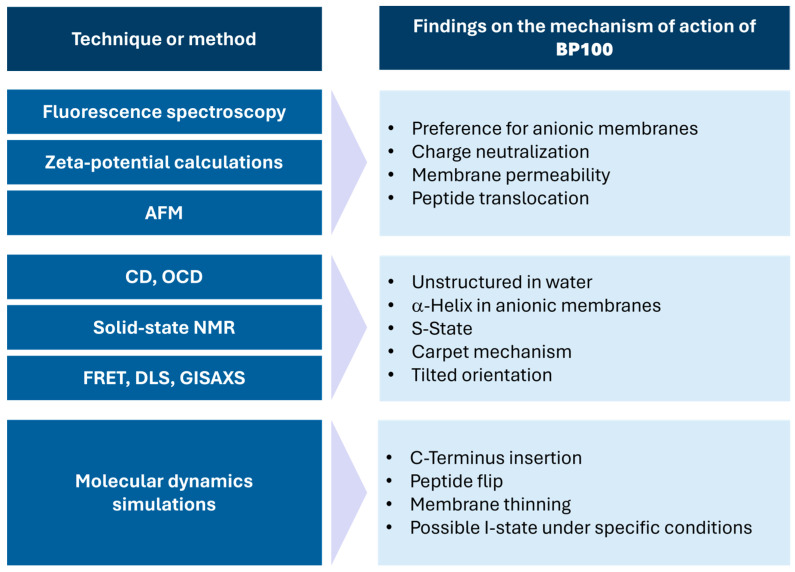
Summary of the main findings on the mechanism of action of BP100 grouped according to the method employed. AFM: atomic force microscopy; CD: circular dichroism; OCD: oriented circular dichroism; NMR: nuclear magnetic resonance; FRET: Förster resonance energy transfer; DLS: dynamic light scattering; GISAXS: grazing incidence small angle X-ray scattering.

The mechanistic studies reported for BP100 are discussed in the following sections classified according to the main method used. The most relevant results obtained using the different techniques are summarized in Figure 1.

### 2.1. Mechanistic Studies Using Biophysical Analysis

Upon the identification of BP100 from a library of linear undecapeptides, we performed preliminary studies by circular dichroism (CD) that revealed that it was unstructured in water while it adopted a partial α-helical conformation in 50% trifluoroethanol (TFE) [23]. In 2009, in collaboration with the group of M. Castanho, we published the first report on the mechanism of action of BP100 [24]. This work focused on understanding this mechanism of action by means of photophysical techniques. Taking advantage of the intrinsic fluorescence of BP100 due to the tyrosine residue present in its sequence, the binding affinity of this peptide as well as its damaging effects on phospholipid bilayers were analysed. This study revealed the importance of the interplay of three factors: charge neutralization, membrane permeabilization, and peptide translocation. First, it was observed that BP100 did not aggregate in aqueous solution, which facilitated the studies on its interaction with model membranes. In particular, in neutral models that mimicked the outer leaflet of mammalian membranes, composed of 100% 1-palmitoyl-2-oleoyl-*sn*-glycero-3-phosphocholine (POPC) and POPC/cholesterol, fluorescence followed a hyperbolic-like pattern. In addition, moderate partition constant (*K*p) values were obtained due to hydrophobic and van der Waals interactions between these neutral systems and the noncharged residues of BP100. Moreover, cholesterol did not improve this interaction, which could be related to the low toxicity of BP100 in mammalian cells. In contrast, in anionic 1-palmitoyl-2-oleoyl-*sn*-glycero-3-(phosphor-rac-(1-glycerol)) (POPG)/POPC vesicles mimicking bacterial cell membranes, the partition curves deviated from a hyperbolic-like pattern at low lipid concentrations, and the *K*p values were found to be higher than those determined in the neutral models. These results pointed out to membrane saturation and to an expected preference of the cationic sequence of BP100 for negatively charged membranes. At the saturation point, electroneutrality was predicted to be reached, because 5.6 phospholipids were calculated for each BP100 sequence (net charge of +6). The occurrence of electroneutrality was also supported by the vesicle aggregation induced by this peptide at membrane saturation. Additionally, upon saturation, the tyrosine residue of BP100 maintained its location in the membrane, near the centre of the bilayer, which could contribute to the destabilization of the membrane. Moreover, it was found that BP100 induced membrane permeabilization, with the rate of permeabilization depending on the peptide concentration. After saturation, the high degree of leakage induced by BP100 pointed out a severe membrane damage or lysis. In the same line, the occurrence of translocation at both high and low peptide-to-lipid (P/L) ratios evidenced that BP100 was able to cross the membrane and its activity did not exclusively rely on the extracellular level.

The importance of neutrality on the antimicrobial activity of BP100 was evidenced by the group of M. Castanho [25]. They analysed the process of neutralization in the interaction between BP100 and the Gram-negative bacteria *Escherichia coli* by means of zeta-potential calculations. These authors showed that the zeta-potential was negative in the absence of peptide and gradually grew with increasing BP100 concentration until neutrality was reached. The peptide concentration at which a zeta-potential of zero was achieved was found to match the minimum inhibitory concentration (MIC). Moreover, atomic force microscopy (AFM) studies revealed that the damage of BP100 on the *E. coli* membrane was time- and concentration-dependent. It was observed that exposure of *E. coli* to BP100 for short times (0.5 h) or to BP100 concentrations below the MIC caused minor damage to the cell membrane. Longer times (≥2 h) and higher BP100 concentrations (≥MIC) led to an increase in the surface corrugation, resulting in the membrane disruption with the formation of vesicle-like structures and alterations in the surface roughness. After permeabilization of the outer membrane and cell envelope, BP100 was able to interact with the negatively charged phospholipids of the bacterial inner membrane, which was associated with membrane saturation [24] and neutralization and finally led to the extensive leakage of intracellular contents, causing *E. coli* cell death.

The tendency of BP100 to adopt an amphipathic α-helical conformation was predicted by I. M. Cuccovia and coworkers through theoretical calculations of the BP100’s hydrophobic moment, and it was further confirmed by CD experiments in the presence of large unilamellar vesicles (LUVs) of phosphatidylcholine (PC) and phosphatidylglycerol (PG) [26]. The authors concluded that the P/L ratio and the PG content were crucial for the interaction of BP100 with lipid-containing membranes. In particular, the results indicated that the α-helical conformation increased when increasing the lipid concentration. Moreover, this peptide caused changes on the hydrodynamic diameter and zeta-potentials of PC:PG LUVs, and vesicle aggregation was suggested. It was also found that BP100 induced leakage of PC:PG LUVs. An increase in the P/L molar ratio led to an increase in the leakage and the BP100 concentration necessary to induce leakage of PC:PG LUVs decreased when increasing the PG content. The leakage rates at higher ionic strength were slower than at low ionic strength, which meant that the interaction between BP100 and negatively charged membranes decreased at high ionic strength. The effect of BP100 on giant unilamellar vesicles (GUVs) of POPC and POPC:POPG was also studied using phase contrast optical microscopy, and it was observed that it depended on the charge of the membranes. BP100 promoted the bursting of POPC:POPG GUVs. In contrast, it caused the permeabilization of POPC GUVs but not their disruption. Taking into account all these results, the authors hypothesized a possible mechanism for BP100. Electrostatic interactions with negatively charged lipids played a crucial role in the stabilization of the α-helical conformation of BP100 as well as in the binding with membranes, which increased at higher PG content. At low P/L ratios and low PG content, BP100 could induce the destabilization of the lipid bilayer leading to membrane thinning and a subsequent gradual leakage of membrane inner contents. At higher P/L ratios, it induced an instantaneous loss of inner contents from liposomes. This rapid membrane disruption is proposed to take place through a carpet-like mechanism which is the result of a sequence of events, including lipid clustering, peptide aggregation on the membrane surface, and the formation of peptide–lipid patches leading to membrane disruption.

In a study by the groups of A. Ulrich and M. Castanho [27], it was concluded that the mechanism of action of BP100 was more similar to that of 21–23 amino acid long and pore-forming peptides from the magainin family than to that of the cell-penetrating peptide (CPP) TAT. More specifically, its behaviour resembled that of non-natural magainin-related peptides, such as MSI-103. The common properties of BP100 with MSI-103 were the following: (i) a good uptake into HeLa cells at concentrations lower than 4 µM, probably by endocytosis, as shown by fluorescence microscopy; and (ii) a high fusogenic activity of anionic vesicles, as evidenced by Förster resonance energy transfer (FRET) spectroscopy. Moreover, BP100 induced considerable vesicle leakage, which depended on the lipid composition and the thickness of the lipid bilayer, as well as on the peptide concentration. The leakage was lower than that of the magainin peptides, but among them, it was more similar to that of MSI-103. Concerning TAT, BP100 only resembled this CPP in its inability to cluster anionic lipids. This work reinforced the hypothesis that BP100 is able of damaging the negative membranes through a mechanism that most likely does not involve pore formation.

### 2.2. Mechanistic Studies Using NMR Spectroscopy

**Figure 2:**
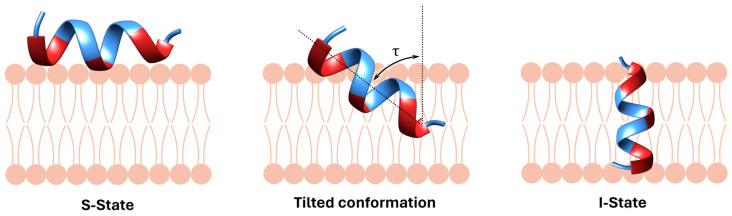
Schematic representation of an S-state, a tilted conformation, and an I-state.

To gain insight into the mechanism of action of BP100, the group of A. Ulrich studied its orientation when interacting with lipid bilayers (1,2-dimyristoyl-*sn*-glycero-3-phosphatidylcholine (DMPC)/1,2-dimyristoyl-*sn*-glycero-3-phosphatidylglycerol (DMPG)) by means of oriented circular dichroism (OCD) and solid-state ^19^F and ^15^N-NMR experiments [28]. They reported for the first time that BP100 adopted a surface-bound state orientation (S-state) (Figure 2). Six BP100 analogues were prepared by replacing each of the uncharged residues with a 3-(trifluoromethyl)-L-bicyclopent-[1.1.1]-1-ylglycine (CF_3_-l-Bpg) moiety, together with a derivative incorporating Leu^8^ labelled with ^15^N, and two analogues bearing both modifications. Results showed that the incorporation of CF_3_-L-Bpg in BP100 did not influence either the antibacterial activity or its α-helical conformation. CD revealed that although being completely unstructured in aqueous media, BP100 and the CF_3_-L-Bpg analogues adopted an α-helical conformation in the presence of anionic vesicles. These results were in agreement with the preliminary ones carried out in TFE [23]. The helix content was of 61% in the case of BP100 and around 75% for the analogues, suggesting that one or two amino acids remained unfolded. OCD and solid-state ^15^N-NMR pointed out that BP100 adopted a S-state in DMPC/DMPG bilayers, with the axis of the α-helix almost parallel to the membrane (tilt angle τ close to 90°). This orientation did not change by varying the P/L ratio, as previously observed for other antimicrobial peptides, such as magainin. Interestingly, BP100 did not aggregate, remaining in a monomeric state upon interaction with the membrane and oligomerization was not observed even at high P/L ratios. The ^19^F-NMR study with the CF_3_-L-Bpg-containing analogues was complemented with the OCD and ^15^N-NMR results, the overall data being analysed through a dynamical model that considered fluctuations of the τ and ρ angles. An azimuthal angle ρ of 160° was found, which indicated that the charged side chains of the lysine residues of BP100 were placed facing the external part of the membrane. Data also suggested that the amidated C-terminus of BP100 might be slightly more inserted into the membrane than the charged N-terminus, and that this peptide exhibited considerable mobility in the membrane with substantial fluctuations in the azimuthal angles. On the basis of these results, the authors predicted a carpet-like mechanism for BP100.

With the aim of obtaining information regarding BP100’s alignment, structure, and dynamics when bound to different membranes, the group of A. Ulrich studied the behaviour of the above-mentioned six BP100 analogues containing a CF_3_-l-Bpg moiety in model membranes and in biomembranes via a multinuclear solid-state NMR study [29]. Bilayers 1,2-dilauroyl-*sn*-glycero-3-phosphatidylcholine (DLPC), 1,2-dierucoyl-*sn*-glycero-3-phosphatidylcholine (DErPC), 1,2-diphytanoyl-*sn*-glycero-3-phosphocholine (DPhPC), and mixtures of DMPC/DMPG and of DMPC/cholesterol differing in charge, fluidity, and thickness were employed as model membranes. *Micrococcus luteus* and erythrocyte ghosts were used to exemplify prokaryotic and eukaryotic membranes, respectively. ^19^F-NMR experiments showed that all six analogues displayed the same secondary structure, mobility, and orientation in the different membrane systems irrespective of their lipid composition, charge, or thickness. From the ^31^P-NMR signal of the phospholipids, it was inferred that BP100 did not significantly alter the bilayer, precluding a mechanism involving the formation of pores or micelles. ^2^H-NMR analysis pointed out that the addition of BP100 to deuterated DMPC/DMPG bilayers resulted in membrane thinning and destabilization. It is worth noting that although the thinning effect was described in both anionic (DMPG) and zwitterionic (DMPC) lipids, in the first one, it was slightly more significant. These results were compatible with the previously reported carpet mechanism [28] in which BP100 would be located within the headgroup region of the bilayer being embedded with the helix axis parallel to the membrane surface, leading to membrane thinning.

A. Ulrich and coworkers further confirmed the induction of membrane thinning by BP100 using solid-state ^2^H-NMR and grazing incidence small angle X-ray scattering (GISAXS) on oriented phospholipid bilayers [30]. Solid-state ^2^H-NMR was used to determine the hydrophobic thickness of the bilayer whereas GISAXS allowed the measurement of the bilayer–bilayer repeat distance. Results from these experiments showed that BP100 reduced these two parameters. These authors also studied other membrane-active peptides, namely PGLa, magainin 2, gramicidin S, and TisB, and they observed a correlation between peptide orientation in the bilayer and the parameters related to membrane thickness. Thus, magainin 2, gramicidin S, and BP100 that bound to the outer layer of the membrane with the helix axis parallel to the bilayer surface (S-state) were more effective at inducing membrane thinning than those inserted in the bilayer, such as TisB.

A. Ulrich and coworkers also pointed out that the spontaneous curvature of the lipidic bilayer can have an influence on the orientation of BP100 in the bilayer and analysed its alignment in oriented membranes of different composition by means of solid-state ^2^H-NMR [31]. In order to overcome the problems associated with the use of the bulky and hydrophobic CF_3_-l-Bpg moiety that only allowed the labelling of the hydrophobic face of BP100, they performed an alanine scan by replacing each residue of BP100 with 3,3,3-^2^H_3_-Ala (Ala-*d*_3_). The analysis of the eleven Ala-*d*_3_-labelled analogues by solid-state ^2^H-NMR in oriented membranes of different composition gave more accurate information about the orientation and dynamics of BP100 in the membrane. CD and ^2^H-NMR data showed that BP100 formed a very stable α-helix when bound to membranes. In addition, the ^2^H-NMR experiments revealed that the helix tilt angle and its dynamical behaviour depended on the peptide concentration and on the spontaneous curvature of the bilayers. In bilayers with a negative spontaneous curvature, such as POPC/POPG (3:1), BP100 lied essentially flat on the bilayer surface irrespective of the concentration. In contrast, in DMPC/DMPG and specially in DMPC/DMPG/1-myristoyl-2-hydroxy-*sn*-glycero-3-phosphatidylcholine (lyso-MPC) (1:1:1) with a positive spontaneous curvature, BP100 oriented flat on the membrane surface at low concentrations, whereas it adopted a tilted orientation at high concentrations, and it showed increased mobility (Figure 2). It was also demonstrated that the C-terminus of BP100 became inserted more deeply into the hydrophobic core of the bilayer, probably with the charged groups of the lysine residues snorkelling to remain in a polar environment. These findings reinforced their previous results based on a carpet-like mechanism involving the binding of BP100 to the membrane surface, followed by its partial insertion, via the C-terminus, into the bilayer core in a highly mobile and monomeric state [28,29,30]. This interaction would cause membrane permeability leading to bacterial cell death.

In addition, the influence of each residue in BP100 on the biological activity was evaluated by screening the antibacterial activity and haemolysis of the eleven alanine-containing analogues. It was observed that none of the lysine residues was essential for the antibacterial activity and that, in contrast, a significant decrease in the activity occurred when one of the hydrophobic residues was replaced. These results revealed the importance of the hydrophobic face of the α-helix of BP100 on the biological activity. Regarding haemolysis, no clear correlation was obtained; however, two peptides with an improved therapeutic index were identified, resulting from the replacement of Lys^2^ or Lys^9^ by an alanine.

The effect of BP100 on membranes of intact Gram-positive *Bacillus subtilis* cells was studied using static ^2^H-NMR [32]. Towards this aim, *B. subtilis* cells with deuterated acyl chains in the membrane were prepared. It was observed that the addition of BP100 caused significant changes in the ^2^H-NMR spectra including a reduction in the sharp edges and an increase in intensity at narrower splittings. In addition, these spectral changes were concentration-dependent, with more pronounced effects observed at higher peptide-to-lipid ratios. These results could be attributed to an increase in the disorder of the acyl chains and a perturbation of the membrane. The authors also suggested the importance of the nonlipid components in the bacterial cell, such as the peptidoglycan layer and/or the lipoteichoic acids, in modulating the interactions of BP100 with the membrane.

From the above works, it can be excerpted that BP100 binds flat to the membrane surface in an S-state, or at most slightly titled, and a carpet mechanism is postulated. Its α-helix conformation and short length excludes that the peptide could form pores. However, in 2023, A. Ulrich and coworkers [33] reported that under specific conditions, this peptide was also able to adopt a stable I-state by extending its charged lysine side chains to both sides of the membrane (Figure 2). This conclusion resulted from an extensive study on the temperature dependence of the orientation of BP100 in lipid bilayers by solid-state ^19^F, ^15^N and ^2^H-NMR. From these experiments, it was observed that at high temperatures, BP100 predominantly assumed an S-state slightly tilted with high mobility and that at temperatures close to the gel-to-liquid crystalline phase transition (*T*m), it adopted the I-state. This state was stable in thin bilayers at low peptide concentrations, while higher peptide concentrations were necessary in thicker membranes. Other factors influenced the transition from the S-state to the I-state, such as the membrane composition. In particular, the presence of lysolipids favoured a transmembrane orientation even at high temperatures.

### 2.3. Mechanistic Studies Using Molecular Dynamics Simulations

The first work focused on the in silico study of the mechanism of action of BP100 was published by Castanho and coworkers in 2012 [34]. Specifically, as a first approach, Brownian dynamics (BD) simulations with a coarse-grained peptide model of BP100 in an environment mimicking mammalian and bacterial cell membranes were employed to predict the structure and behaviour of this peptide. An α-helical structure in an amphipathic arrangement was predicted when the peptide was confined in POPC, POPC:cholesterol, and POPC:POPG membrane models. This α-helical conformation was confirmed by the Cα dihedral angles of each amino acid of BP100, the opposite distribution of the charged and the hydrophobic residues along the helix, and the distance between the N- and C-terminal residues. During the 1 μs simulations, BP100 showed a notable lateral diffusion and displayed a tendency to distribute itself without any specific orientation, being placed halfway across the membrane leaflet. It was also concluded that in negatively charged membranes, the orientation of BP100 varied less than in zwitterionic membranes, and its lateral diffusion was lower. Taking into consideration these observations and the small length of BP100, a mechanism involving pore formation was discarded. In contrast, a carpet- or detergent-like mechanism was more likely to be responsible for the mode of action of this peptide. All these results supported previous reports describing the neutralization and saturation of anionic bacterial model membranes and the *E. coli* membrane when exposed to BP100 at concentrations equivalent to and greater than MIC values [24,25].

Ulrich and coworkers observed that long sampling timescale (>1.5 µs) molecular dynamic (MD) simulations were in accordance with BP100 experimental data [35]. These simulations were carried out in a DMPC bilayer in the surface-bound S-state at 35 °C, using the CHARMM/AA-lipid force field and starting from a fully helical conformation. First, the peptide inserted the C-terminus into the membrane, the tilt angle τ being >150°. Over the 1.5 µs simulation, the tilt angle τ was 109° and, after 1.5 µs, it decreased considerably due to the unfolding of the N-terminal lysines, remaining at 97° until the end of the simulation. Moreover, the helicity of the peptide in the second simulation phase >1.5 µs was in accordance with that determined by CD for the BP100 analogues bearing a CF_3_-L-Bpg. A comparison of the results obtained before and after 1.5 µs suggested that the latter were more in line with the experimental data. Based on this, the authors concluded that the initial simulation conditions could influence the behaviour of peptides in short MD simulations while long-term simulations were more likely to match experimental data.

**Figure 3:**
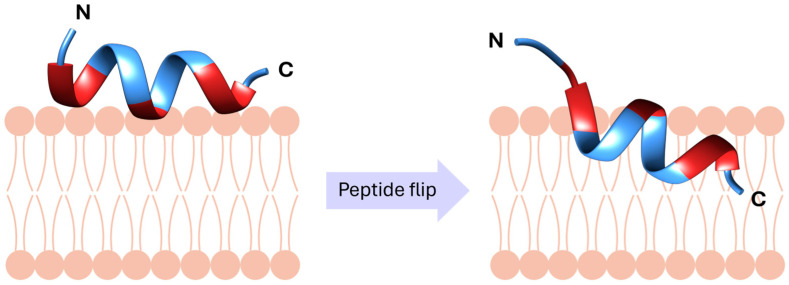
Schematic representation of a peptide flip. Polar residues are marked in red and apolar in blue.

I. M. Cuccovia and coworkers [36,37] furthered the understanding of the interaction of BP100 with membranes through MD simulations and they described for the first time that after an initial electrostatic interaction with the membrane, BP100 flipped, a step that could be relevant for the carpet mechanism of this peptide (Figure 3). The MD simulations were performed with longer sampling times (more than 25 µs) than those previously described by Wang et al. [35]. They studied the secondary structure and behaviour of BP100 in solution and in bilayers of zwitterionic 1,2-dipalmitoyl-*sn*-glycero-3-phosphatidylcholine (DPPC) and anionic 1,2-dipalmitoyl-*sn*-glycero-3-phosphoglycerol (DPPG) lipids as well as in two mixed membranes DPPC:DPPG (1:1). One mixed membrane was composed of randomly distributed lipids and the other contained a DPPG raft in the middle of the monolayers. As expected, BP100 adopted a random coil conformation in solution, whereas in lipid bilayers, BP100 folded into an α-helix. It was determined that the percentage of α-helix was proportional to the content of PG, being higher in anionic DPPG bilayers (73%) than in the zwitterionic DPPC ones (38%). In all the simulations, the α-helix formed between residues Lys^5^ to Lys^9^ displayed high stability, suggesting that it played an important role in the formation of this secondary structure upon peptide interaction with the membrane. Interestingly, during the simulations, they observed that after an initial electrostatic interaction between the negatively charged membrane and the positively charged lysine residues, BP100 rotated. This rotation prompted the insertion of the hydrophobic face of BP100 into the hydrophobic region of the membrane. They termed this dynamic behaviour as peptide flip, as this was the first report on this phenomenon in MD simulations of peptide/membrane interactions, and it could be a crucial step of the carpet mechanism of BP100 as well as of other cationic antimicrobial peptides. After the flip, peptide dehydration as well as an increase in the apolar peptide/lipid contacts were observed, pointing out that the apolar residues of BP100 inserted deeper into the bilayer. These results suggested that the hydrophobic residues of BP100 also played a key role in its initial interaction with membranes. It was also determined that BP100 promoted the clustering of both anionic and zwitterionic lipids, but in the presence of the latter, it lost the secondary structure, which was attributed to the presence of positively charged choline groups. All these data were consistent with the higher activity of BP100 against bacteria versus mammalian cells. Accordingly, BP100 would interact electrostatically with the negatively charged bacterial membrane which would promote α-helix formation, leading to peptide flip and insertion into the hydrophobic core of the membrane. In contrast, the zwitterionic lipids present in mammalian cells would not favour the formation of the α-helix and the peptide flip, resulting in low activity.

I. M. Cuccovia and coworkers also determined that upon binding, BP100 influenced membrane thickness, surface curvature, lipid acyl-chain order parameters, lateral lipid diffusion, and membrane hydration, and that these effects depended on the local membrane composition and on the lipid distance to the membrane-bound peptide [37]. They observed that BP100 caused significant membrane thinning, especially in membranes containing anionic lipids, and that this effect was more important in the lipids closer to the peptide. In addition, BP100 induced a high local negative curvature and increased the conformational freedom of the lipids’ chain in DPPG bilayers and in DPPC/DPPG membranes containing a DPPG raft, especially after the peptide flip. Regardless of the membrane composition, BP100 decreased the lateral lipid diffusion and, as in the other membrane properties, this disturbance was higher in lipid groups in close proximity to the peptide. Peptide binding and flip favoured water penetration and transmembrane water transport in DPPG and in DPPG-containing lipid rafts compared to membranes in which a full peptide flip did not occur.

Furthermore, molecular dynamics served to assess the feasibility of the I-state conformation determined by NMR (Figure 2) [33]. To this end, S-state and I-state simulations were conducted in a DMPC lipid bilayer. In the S-state simulation, after 1.5 μs, the initial fully helical structure of BP100 reduced its helicity to approximately 80%, the N-terminal Lys^1^ and Lys^2^ becoming unfolded. During this simulation, BP100 remained almost flat on the membrane with a helix tilt angle close to 90° with small fluctuations from 10° to 20°. In the I-state simulation, the tilt angle was 156°, indicating an almost vertical transmembrane conformation in the DMPC bilayer with the positive lysine side chains snorkelling on both sides of the membrane pulling water molecules into the lipid bilayer. Although both orientations were found to be possible for BP100, there seemed to be a large energetic barrier to switch from one to the other. In any case, all these results raised the possibility that the mechanism of action of BP100 may involve a stable transmembrane orientation.

Table 1 includes the methods or techniques employed in all the works described in Section 2 to elucidate the mechanism of action of BP100. It also details the system used to perform the experiments as well as the main findings achieved. They are listed according to their appearance in the text.

## 3. Mechanistic Studies on BP100 Derivatives

The groups of M. Castanho, P. Gameiro, and I. M. Cuccovia synthesized BP100 analogues with the aim of analysing the effect of incorporating structural modifications on the mode of action (Table 2) [38,39,40,41]. In particular, M. Castanho and coworkers studied the activity against Gram-negative and Gram-positive bacteria as well as the mechanistic features of two BP100 analogues: R-BP100 (RRLFRRILRYL-NH_2_) and RW-BP100 (RRLFRRILRWL-NH_2_) [38]. Both analogues were designed by replacing the lysine residues in BP100 by arginine and, additionally, in the case of RW-BP100, Tyr^10^ was substituted by a tryptophan. These analogues displayed higher activity than BP100 against Gram-negative bacteria (*E. coli*, *Klebsiella pneumoniae*, and *Pseudomonas aeruginosa*) and, unlike BP100, they were also active against Gram-positive bacteria (*Staphylococcus aureus, Streptococcus pneumoniae*, and *Enterococcus faecium*), RW-BP100 being the most active one. In addition, these two analogues were more haemolytic than BP100. Mechanistic studies by AFM imaging evidenced that the three peptides exerted similar effects on the membrane surface of *E. coli*, but the effect produced by R-BP100 and RW-BP100 on *S. aureus* membrane was more important than that induced by BP100. The three peptides had more affinity for negative membranes than zwitterionic ones, as shown by surface plasmon resonance (SPR), changes in the dipolar potential of POPC/POPG and POPC membranes, vesicle aggregation, leakage from vesicles, and fluorescence emission quantum yield. A higher concentration of BP100 was required to cause the disruption of the cell envelope of *S. aureus* due to its lower affinity for lipoteichoic acid (LTA), which is the main negatively charged component present in the envelope of Gram-positive bacteria, in comparison to its binding with lipopolysaccharides (LPS) present in Gram-negative bacteria. The interaction of BP100 and the two analogues with the membrane was not restricted to an external binding with its anionic components, but they inserted into the membranes, especially in negatively charged membranes, with RW-BP100 being more deeply inserted, probably due to the presence of a tryptophan in its sequence. In this process, these peptides adopted an amphipathic α-helix that facilitated the electrostatic interaction between the cationic residues of the sequence at one side of the helix and the headgroups of phospholipids, as well as between the nonpolar residues placed on the opposite side of the helix with the hydrophobic components of the bilayer. Based on this, the authors suggested that the mechanism of action of these peptides would involve their binding to the negatively charged membranes, followed by their insertion into the lipid bilayer and the subsequent disruption and final permeabilization of the membrane. It was argued that the substitution of the lysine residues with arginine favoured the membrane-binding affinity as compared with BP100, probably due to the guanidinium side chain’s ability to simultaneously form stronger hydrogen bonds and cation–π interactions with lipid membranes. Arginine residues also conferred activity against Gram-positive bacteria. In the case of RW-BP100, the presence of a tryptophan resulted in higher hydrophobicity and favoured its insertion into the membrane by enhancing cation–π interactions.

Later, the group of P. Gameiro [39,40] studied the antibacterial activity and the biophysical properties of W-BP100 (WKKLFKKILKYL-NH_2_), incorporating an additional tryptophan residue at the N-terminus. The Edmunson wheel projection of W-BP100 showed that this tryptophan was located at the hydrophobic face and therefore, similarly to BP100, this analogue had an amphipathic character. As previously reported for RW-BP100, the incorporation of a tryptophan resulted in an increase in the activity against Gram-positive bacteria compared to BP100, while the activity against Gram-negative species was similar. Moreover, although the total charge was maintained, this aromatic residue was suggested to promote cation–π interactions with the lysine residues enhancing the peptide structuration and allowing a deeper insertion into the membranes. Mechanistic studies of the interaction of W-BP100 with LUVs were carried out by fluorescence spectroscopy, dynamic light scattering (DLS), and nanoparticle tracking analysis (NTA) experiments. W-BP100 showed a higher *K*p in anionic POPC:POPG (1:1) LUVs than in zwitterionic POPC membranes, and it was able to induce membrane saturation. In addition, while both W-BP100 and BP100 showed reduced accessibility of aromatic residues to the aqueous environment, W-BP100 displayed stronger quenching in the presence of acrylamide, suggesting a better insertion into the membrane. Similarly to BP100, the release of 5(6)-carboxyfluorescein (CF) from anionic vesicles depended on the concentration of W-BP100 and on the P/L ratios, even though it occurred at a higher rate than BP100. In particular, W-BP100 caused a slow and gradual release of CF at low P/L ratios and low peptide concentration, while a more important and fast leakage occurred at high P/L ratios and high peptide concentration. However, in contrast with BP100, at concentrations close to membrane saturation, W-BP100 led to a reduction in the vesicle size and only some aggregation of POPC:POPG (1:1) LUVs was detected. DLS and NTA experiments showed that W-BP100 minimally affected LUVs at low and high P/L ratios, and it only caused an increase in the size of vesicles at intermediate P/L ratios of around 0.07 to 0.12, which was attributed to vesicle aggregation. At the P/L ratio at which aggregation took place, charge neutralization occurred, and the α-helical conformation of W-BP100 was maintained. According to these results, the monitoring of membrane fusion by FRET revealed that W-BP100 promoted membrane fusion at intermediate P/L ratios, which could also be associated with vesicle aggregation. Finally, confocal microscopy showed that the effects of W-BP100 in fluorescent-labelled GUVs also depended on the P/L ratio following a similar trend to that observed for the LUVs. Thus, this peptide did not lead to significant alterations at low P/L ratios, while it induced the aggregation of the vesicles at intermediate P/L ratios, and it caused the formation of new lipid structures at high P/L ratios. These results suggested a detergent-like mechanism, ultimately leading to lipid remodelling and the formation of stable lipid aggregates. All these results showed that the addition of a single tryptophan amino acid to BP100 led to an improvement of the activity and to results comparable to those obtained when substituting all the lysine residues by arginines and Tyr^10^ by a tryptophan [38].

I. M. Cuccovia and coworkers [41] synthesized two analogues of BP100 with the aim of evaluating the influence on the conformation and the biological activity of increasing the hydrophobicity and of extending the α-helical structure. Regarding the former, they prepared an analogue incorporating an alanine residue and a hexadecyl chain at the C-terminus of BP100 (BP100-Ala-NH-C_16_H_33_). The latter analogue contained at the N-terminus the cyclic peptide *cyclo(1-4)*-D-Cys^1^, Ile^2^, Leu^3^, Cys^4^, which included a disulfide bond between residues D-Cys^1^ and Cys^4^ (*cyclo*(1-4)-cILC-BP100). The binding of these two analogues to LUVs of different composition was examined using fluorescence and CD spectroscopies. Results showed that they bound to lipid vesicles containing 30–50% POPG with higher affinity and lesser surface charge dependence than BP100, which was attributed to their higher hydrophobicity. It was determined that the lipopeptide analogue (BP100-Ala-NH-C_16_H_33_) exhibited a similar α-helix conformation than BP100 and a C-terminus more deeply buried into the membrane. In contrast, the derivative containing the cyclic moiety (*cyclo*(1-4)-cILC-BP100) adopted a conformation that was partly α-helix and partly β-turn and had an N-terminus more strongly anchored into the membrane. Regarding the mechanism of action, it was demonstrated that at high peptide concentrations, they displayed a similar carpet mechanism to BP100. At low concentrations, results suggested that these peptides perturbated the membrane organization, probably causing thinning and resulting in an increase in the permeability. The activity of BP100-Ala-NH-C_16_H_33_ and *cyclo*(1-4)-cILC-BP100 against Gram-negative and Gram-positive bacteria was similar to that of the parent peptide BP100, but they were more haemolytic.

**Table 2:** Summary of the main findings on the mechanism of action of BP100 derivatives.

BP100 Derivative	Method or Technique	System	Main Findings	Refs.
RRLFRRILRWL-NH_2_ (RW-BP100) RRLFRRILRYL-NH_2_ (R-BP100)	AFM SPR Fluorescence spectroscopy CD	LUVs, SUVs: POPC POPC:POPG (1:1, 4:1)	Incorporation of a Trp and Arg residues confers activity against Gram-positive bacteria. Trp favours membrane insertion and Arg membrane binding affinity.	[38]
WKKLFKKILKYL-NH_2_ (W-BP100)	Zeta-potential calculations CD Fluorescence spectroscopy and microscopy DLS NTA FRET	LUVs: POPC:POPG (1:1, 3:1) POPC POPE:POPG (1:1) GUVs: POPC:POPG (1:1)	Incorporation of a Trp at the N-terminus enhances the antibacterial activity. At intermediate P/L ratios, it promotes charge neutralization and vesicle aggregation. At high P/L ratios, it causes the formation of new lipid structures.	[39,40]
BP100-Ala-NH-C_16_H_33_ *cyclo*(1-4)-cILC-BP100 (Figure 4)	CD Fluorescence spectroscopy Zeta-potential calculations	LUVs POPC POPC:POPG (7:3, 1:1, 3:7) GUVs: POPC:POPG (7:3)	They exhibit similar antibacterial activity to BP100 and are more haemolytic. They display a similar carpet mechanism to BP100.	[41]

**Figure 4:**
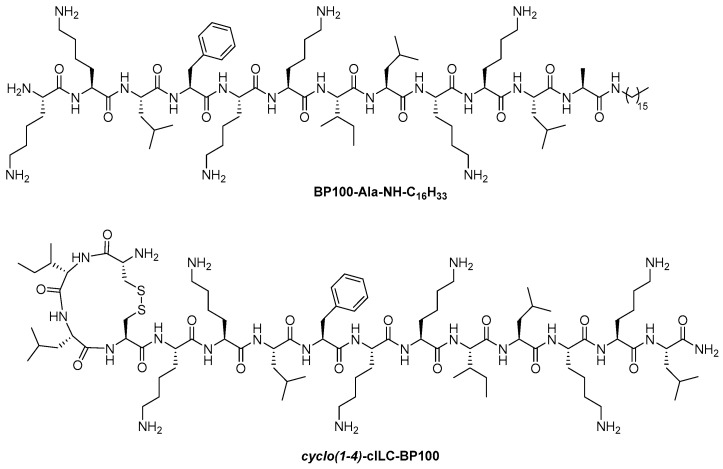
Structures of BP100-Ala-NH-C_16_H_33_ and *cyclo*(1-4)-cILC-BP100.

## 4. Final Remarks and Conclusions

The mechanism of action of BP100 has been studied using spectroscopic techniques (fluorescence, NMR, CD, OCD, FRET), biophysical studies, microscopy, and MD simulations which have been carried out in anionic, zwitterionic, and mixed membranes, in vesicles (LUVs and GUVs), and in bacteria. These studies have allowed researchers to determine the structural changes that occur in both BP100 and the membrane upon interaction. The spectroscopic experiments, microscopy, and biophysical analysis have revealed that BP100 exhibits a preference for anionic membranes and that its mechanism of action primarily involves charge neutralization and membrane permeabilization. Membrane neutralization occurs at the MIC and at this concentration, BP100 causes an increase in the surface corrugation, leading to membrane disruption with vesicle formation and surface roughness alteration. Regarding the peptide conformation, in anionic membranes, BP100 transitions from an unstructured state in water to an α-helix with the axis parallel to the surface (S-state). In addition, under specific conditions, BP100 can adopt an I-state with the lysine side chains extended to both sides of the membrane. It has been determined that the tilt angle and mobility of BP100 depend on the peptide concentration and the spontaneous curvature of the bilayers. In the case of bilayers with a negative spontaneous curvature, the orientation of BP100 does not depend on the concentration and it remains flat on the surface. In contrast, in bilayers with a positive spontaneous curvature, BP100 shows higher mobility, and at high concentrations, it adopts a tilted orientation with the C-terminus more inserted into the bilayer and with the side chains of the lysine residues snorkelling to remain in a polar environment. The P/L ratio also plays an important role in the interaction of BP100 with membranes. It induces bilayer destabilization and gradual leakage at low P/L ratios, while higher P/L rations lead to rapid membrane disruption and therefore, to an instantaneous leakage. All this experimental evidence together with the small length of this peptide suggest that a carpet-like mechanism is more likely to occur, and that it is similar to the one described for magainin-related peptides.

**Figure 5:**
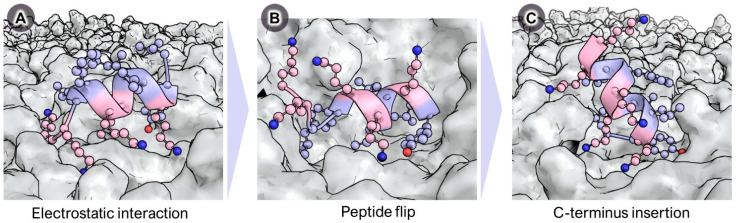
Main steps of the mechanism of action of BP100 (Lys are marked in pink and hydrophobic residues are in purple).

These results are supported by the MD simulations, which have gone a step further in the understanding of the mechanism of action of BP100. These simulations have shown that after the electrostatic interaction (Figure 5A) with the membrane, BP100 flips (Figure 5B). This rotation could be a key step of the carpet mechanism because it favours the insertion of the hydrophobic face of the peptide into the hydrophobic core of the membrane. In fact, from MD simulations, it has also been proposed that BP100 adopts an almost vertical transmembrane orientation with the lysine side chains snorkelling on both sides of the membrane and with the C-terminus inserted into the membrane (Figure 5C). Thus, both cationic (electrostatic interaction) and hydrophobic (peptide insertion) residues are essential for the mode of action of BP100. Moreover, the peptide flip induces local membrane thinning and a slow lipid diffusion and favours water penetration, these effects being more prominent in membranes composed of anionic lipids.

It should be highlighted that all these data are consistent with the biological activity profile reported for BP100. In particular, this peptide is highly active against Gram-negative bacteria while having low haemolytic activity. According to the postulated carpet-like mechanism, the anionic character of the bacterial membrane would facilitate the electrostatic interaction with the cationic lysine residues of BP100, promoting the adoption of an α-helical conformation, followed by a peptide flip and insertion into the membrane. In contrast, the mammalian cell membrane is rich in zwitterionic lipids, which hamper the α-helix formation and the subsequent peptide rotation, resulting in a low haemolytic activity.

Several BP100 derivatives have been synthesized with the aim of analysing the influence of the incorporation of arginine and/or tryptophan residues on the mechanism of action of BP100. All these analogues followed a similar carpet-like mechanism to that of the parent peptide. The cationic guanidinium side chain of arginine increases the electrostatic interaction with the anionic membrane, while the high hydrophobicity of tryptophan favours the membrane insertion.

The cumulative findings described herein provide important information about the structural properties of BP100 and contribute significantly to unravelling its mechanism of action. All these data provide the basis for future studies towards the prediction, rational design, and development of new peptides derived from BP100 with improved biological properties. In addition, this knowledge offers valuable insights towards the design of new and efficient antimicrobial peptides.

## Figures and Tables

**Table 1:** Summary of the main findings on the mechanism of action of BP100.

Method or Technique	System	Main Findings	Refs.
Fluorescence spectroscopy Zeta-potential calculations	LUVs: POPC POPC:cholesterol (2:1) POPG:POPC (2:1, 4:1)	Preference for anionic membranes. Three key factors in the BP100 mechanism of action: charge neutralization, membrane permeabilization, and peptide translocation.	[24]
Zeta-potential calculations AFM	Bacterium *E. coli*	Membrane neutralization is reached at MIC. Membrane disruption: alteration in the surface roughness and vesicle formation.	[25]
CD NMR Zeta-potential calculations Dynamic light scattering Fluorescence spectroscopy Optical microscopy	LUVs: POPG POPC:POPG (3:1, 1:1, 1:3) GUVs POPC POPC:POPG (7:3)	At low P/L ratios, BP100 causes the destabilization of the bilayer and gradual leakage. At higher P/L ratios, BP100 induces a rapid membrane disruption through a carpet-like mechanism.	[26]
Fluorescence microscopy and spectroscopy FRET spectroscopy	SUVs: POPC:POPG (4:1, 1:1) POPC:cholesterol (7:3) POPC:POPG:cholesterol (8:2:5) POPE:POPG (7:3, 1:1) POPC:POPE:POPS:SM:cholesterol (10:5:2:2:10) DMoPC:DMoPG (1:1) DErPC:DErPG (1:1)	BP100 has a similar mechanism to magainin-like peptides. Its amphipathic α-helix disturbs the membrane causing permeabilization without pore formation.	[27]
CD and OCD Solid-state ^15^N- and ^19^F-NMR	SUVs and oriented bilayers: DMPC:DMPG (3:1)	Unstructured in water but it folds into an α-helix in anionic membranes with an S-state conformation (i.e., with the axis parallel to the surface, tilt angle τ ≈ 90°). Carpet mechanism with the C-terminus slightly inserted.	[28]
Solid-state ^2^H-, ^31^P- and ^19^F-NMR	Oriented bilayers DMPC:DMPG (3:1) DLPC DErPC DMPC:cholesterol Native membranes	BP100 does not significantly alter the bilayer (discarding a mechanism involving the formation of pores or micelles) but affects its thinning (compatible with a carpet mechanism).	[29]
^2^H-solid state NMR GISAXS	Oriented bilayers DMPC	BP100, in the S-state, reduces the bilayer hydrophobic thickness and bilayer–bilayer repeat distance.	[30]
^2^H-solid state NMR CD Alanine scanning	Oriented bilayers POPC:POPG (3:1) DMPC:DMPG (3:1) DMPC:DMPG:lyso-MPC (1:1:1)	The tilt angle and mobility of BP100 depends on the peptide concentration and the spontaneous curvature of the bilayers. In POPC/POPG bilayers (negative curvature), BP100 lies flat with low mobility. In DMPC/DMPG membranes (positive curvature), a tilted orientation is observed with the C-terminus more inserted and the Lys residues snorkelling.	[31]
^2^H-solid state NMR	^2^H-Enriched membrane of bacterium *B. subtilis*	Concentration-dependent spectral changes pointed to a higher perturbation of the membrane at high peptide-to-lipid ratios.	[32]
CD and OCD ^19^F-, ^15^N- and ^2^H-solid state NMR MD simulations	Oriented bilayers: DMPC:DMPG (3:1, 1:1, 1:3) DLPC:DLPG (3:1) DLPC DLPC:lyso-LPC (2:1) DMPC DMPC:lyso-MPC (2:1) Model bilayers DMPC	Under specific conditions, BP100 can also adopt a transmembrane I-state with the lysine side chains extended to both sides of the membrane	[33]
Brownian dynamics	Model membranes: POPC POPC:cholesterol POPG:POPC	BP100 adopts an α-helix with notable lateral diffusion and without specific orientation in the membrane.	[34]
MD simulations	Model bilayers: DMPC POPC	Starting from a fully helical conformation, the insertion takes place through the C-terminus with a significant tilt angle.	[35]
MD simulations	Model bilayers: DPPC DPPG DPPC:DPPG (1:1)	After an initial electrostatic interaction, a peptide flip takes place, being responsible for the insertion into the membrane. The peptide flip induces local membrane thinning and a slow lipid diffusion and favours water penetration.	[36,37]

## Abbreviations

AFM	atomic force microscopy
BD	Brownian dynamics
CD	circular dichroism
CF_3_-l-Bpg	3-(trifluoromethyl)-l-bicyclopent-[1.1.1]-1-ylglycine
CPP	cell-penetrating peptide
DErPC	1,2-dierucoyl-*sn*-glycero-3-phosphatidylcholine
DErPG	1,2-dierucoyl-*sn*-glycero-3-phosphatidylglycerol
DLPC	1,2-dilauroyl-*sn*-glycero-3-phosphatidylcholine
DLPG	1,2-dilauroyl-*sn*-glycero-3-phosphoglycerol
DLS	dynamic light scattering
DMoPC	1,2-dimyristoleoyl-*sn*-glycero-3-phosphatidylcholine
DMoPG	1,2-dimyristoleoyl-*sn*-glycero-3-phosphatidylglycerol
DMPC	1,2-dimyristoyl-*sn*-glycero-3-phosphatidylcholine
DMPG	1,2-dimyristoyl-*sn*-glycero-3-phosphatidylglycerol
DPhPC	1,2-diphytanoyl-*sn*-glycero-3-phosphocholine
DPPC	1,2-dipalmitoyl-*sn*-glycero-3-phosphatidylcholine
DPPG	1,2-dipalmitoyl-*sn*-glycero-3-phosphoglycerol
FRET	Förster resonance energy transfer
GISAXS	grazing incidence small angle x-ray scattering
GUVs	giant unilamellar vesicles
LPS	lipopolysaccharides
LTA	lipoteichoic acid
LUVs	large unilamellar vesicles
lyso-LPC	1-lauroyl-2-hydroxy-*sn*-glycero-3-phosphatidylcholine
lyso-MPC	1-myristoyl-2-hydroxy-*sn*-glycero-3-phosphatidylcholine
MD	molecular dynamics
MIC	minimum inhibitory concentration
NMR	nuclear magnetic resonance
NTA	nanoparticle tracking analysis
OCD	oriented circular dichroism
P/L	peptide/lipid ratio
PC	phosphatidylcholine
PG	phosphatidylglycerol
POPC	1-palmitoyl-2-oleoyl-*sn*-glycero-3-phosphocholine
POPE	1-palmitoyl-2-oleoyl-*sn*-glycero-3phosphatidylethanolamine
POPG	1-palmitoyl-2-oleoyl-*sn*-glycero-3-(phosphor-*rac*-(1-glycerol))
POPS	1-palmitoyl-2-oleoyl-*sn*-glycero-3-phosphatidylserine
SM	sphingomyelin
SPR	surface plasmon resonance
SUVs	small unilamellar vesicles
TFE	trifluoroethanol
